# Impact of age on surgical outcomes for world federation of neurosurgical societies grade I and II aneurysmal subarachnoid haemorrhage: a novel prognostic model using recursive partitioning analysis

**DOI:** 10.1007/s10143-024-03067-8

**Published:** 2024-10-30

**Authors:** Motoyuki Umekawa, Gakushi Yoshikawa

**Affiliations:** 1 Department of Neurosurgery, Showa General Hospital, Tokyo, Japan; 2 Department of Neurosurgery, The University of Tokyo Hospital, Tokyo, Japan

**Keywords:** Aneurysm, Age-dependent, Microsurgical clipping, Recursive partitioning analysis, Subarachnoid haemorrhage, World Federation of Neurosurgical Societies grade

## Abstract

This study aimed to evaluate age as a prognostic factor and develop a comprehensive prognostic model for patients undergoing clipping surgery for World Federation of Neurosurgical Societies (WFNS) grade I/II aneurysmal subarachnoid haemorrhage (SAH). We retrospectively investigated 188 patients with WFNS grade I/II SAH who underwent microsurgical clipping at our institute between December 2010 and January 2020. The data of 176 patients (75 with grade I and 101 with grade II) were analysed. Data on patient demographics, aneurysm characteristics, SAH factors, surgical details, and clinical outcomes were collected. Prognostic factors were assessed using bivariate and multivariable logistic regression analyses, and recursive partitioning analysis. Favourable outcomes (mRS 0–2) were observed in 76% of patients. Age, a significant negative prognostic factor in multivariable analysis (odds ratio 0.55, 95% confidence interval 0.40–0.76, *p* < 0.001), was cutoff at 70 years by the receiver operating characteristic curve. Patients aged ≤ 70 years had significantly better outcomes than those aged > 70 years (84% vs. 46%, respectively; *p* < 0.001). Epileptic seizures were significantly associated with poor outcomes in older adults (*p* < 0.001). A prognostic model (favourable, intermediate, and poor) based on age and postoperative adverse events showed significantly different outcomes between age groups (*p* < 0.001). Age was a stronger prognostic factor than WFNS grading for patients with grade I/II SAH undergoing microsurgical clipping. For patients aged ≤ 70 years, precise microsurgeries with fewer complications were associated with favourable outcomes beyond WFNS grade. For older patients, postoperative intensive seizure management may prevent poor outcomes.

## Introduction

Subarachnoid haemorrhage (SAH) has an incidence of 1–20 cases per 100,000 population, with approximately 85% of cases caused by ruptured aneurysms [21, 28]. Rerupture of aneurysms leading to SAH has a mortality rate of 50–60%. Therefore, surgical intervention to prevent rerupture is the primary step for SAH treatment [15, 18, 33]. Treatment approaches can be broadly divided into microsurgery, such as clipping or trapping with bypass, and endovascular treatments, primarily coiling [16, 24, 26]. The frequency of clipping for SAH has decreased with advances in endovascular devices [17, 37], but the rate of recurrence of aneurysms after clipping is lower than that after coiling [2, 5, 9, 40]. Therefore, our institution actively performs microsurgical clipping for aneurysmal SAH from a curative standpoint. However, less invasive endovascular treatments may be selected for older adults or those with poor surgical tolerance, depending on their general condition.

The World Federation of Neurosurgical Societies (WFNS) classification evaluates severity based on the level of consciousness and the presence of neurological symptoms and is widely used as the gold standard due to its correlation with prognosis [34]. While low-grade SAH (WFNS grade I and II) generally has a better prognosis than higher-grade SAH (WFNS grade III-V), outcomes are not always favourable due to factors such as patient background, surgical complications, and adverse events arising from the management of vasospasm or systemic complications [3, 7, 10, 20, 22, 30, 35, 45]. Although various factors influencing prognosis in low-grade SAH have been examined, the evidence remains insufficient. The age at onset has been reported as a prognostic factor for SAH and is simple to evaluate [4, 6, 23, 31, 43]. However, few studies have investigated the effects of age on low-grade SAH outcomes.

Hence, in this study, we aimed to explore the prognostic factors of patients who underwent microsurgical clipping for WFNS grade I and II SAH and develop a simple prognostic model mainly based on patient age of onset.

## Methods

### Participant selection

We collected data on patients who developed SAH from ruptured cerebral aneurysms (including dissecting aneurysms) between December 2010 and January 2020 and underwent open surgery, using an institutional SAH database. Patients whose modified Rankin scores (mRS) were ≥ 3 were excluded. Data regarding patient-, aneurysm-, SAH-, and surgery-related factors and clinical outcomes that were prospectively collected and recorded in the database were retrospectively evaluated. Informed consent was obtained from all participants, and the study was approved by the Institutional Ethics Committee of Showa General Hospital (approval number REC-328).

### Procedures and techniques of SAH management

The treatment protocol for low-grade SAH via microsurgical clipping at our institution was as follows: Initially, digital subtraction angiography (DSA) was performed under sedation and analgesia to determine the treatment strategy. If patients visited within three days of SAH onset, surgery was performed on the same day or the following day. For patients arriving after the fourth day of onset, the timing of surgery depends on the initial DSA findings of vasospasm and the patient’s general condition. Ventricular and cisternal or lumbar drainage is employed postoperatively for ventriculo-cisternal irrigation and intracranial pressure control therapy. Postoperative management of SAH has been described in detail in previous studies [36]. Imaging evaluation primarily involved CT scans to assess SAH distribution until day 3. On day 4, an MRI is performed to evaluate early ischaemic lesions and vascular conditions before the vasospasm. On day 7, DSA was performed to detect cerebral vasospasm and aneurysms. MRI is repeated on day 14 to detect late-stage vasospasms and ischaemic lesions. Additionally, shunt surgery was performed if symptomatic post-SAH hydrocephalus occurred, and appropriate general medical treatment was performed if needed throughout the time course.

### Treatment outcomes and statistical analyses

The median and interquartile ranges (IQR) were calculated for each factor. The primary outcome was the mRS at discharge, with a favourable outcome defined as mRS 0–2. The following complications were assessed in the safety evaluation: emergent additional surgery, vasospasm, delayed cerebral infarction (DCI), epileptic seizures, secondary hydrocephalus (shunt placement excluded from additional surgery), meningitis, and systemic medical complications. Vasospasm was defined as any angiographical vasospasm detected by DSA or magnetic resonance angiography (MRA) with a decrease of ≥ 50% in the cerebral artery diameter relative to the preoperative value. This was judged solely on radiological findings, regardless of symptoms [10]. DCI was defined as symptomatic infarction identified on CT or MRI after excluding surgery-related infarction with any angiographical vasospasm [39].

To create a prognostic model for favourable outcomes, bivariate and multivariable logistic regression analyses were performed for the patient background factors, including age and WFNS grade, SAH factors, and surgical factors. Factors showing significance in the bivariate analysis were included in the multivariable analysis along with age and WFNS grade. Further analysis using factors related to postoperative adverse events was conducted to assess their association with favourable outcomes. In this analysis, factors for multivariable analysis were selected using a stepwise forward selection method with a P-value threshold of < 0.10. A cutoff value for age as a continuous variable was calculated from the receiver operating characteristic (ROC) curve using the Youden index, creating a two-group division for age. Finally, recursive partitioning analysis (RPA) used patient background, including dichotomised age and other significant factors from the bivariate analysis, to develop a three-group prognostic model (favourable, intermediate, poor) for favourable outcomes. P-values of < 0.05 denoted statistical significance. Statistical analyses were performed using JMP Pro 17 software (SAS Institute Inc., Cary, NC, USA).

## Results

### Participant selection

We obtained data on 373 patients who developed SAH due to ruptured cerebral aneurysms (including dissecting aneurysms) between December 2010 and January 2020 and underwent open surgery from an institutional SAH database. Of them, 188 with WFNS grades I and II SAH were identified. After excluding 12 patients whose modified Rankin scores (mRS) were 3 or more, 176 (75 patients with WFNS grade I and 101 with grade II) were included in this study.

### Participant baseline characteristics and surgery for SAH

The median age of the 176 patients with low-grade aneurysmal SAH included in this study was 57 years (IQR, 47–70 years), and females were predominant (68%, Table 1). Hypertension was the most common comorbidity (52%), while diabetes mellitus was rare (4%). Fifteen patients (9%) already had mRS scores of 1 or 2 before SAH onset. For 93% of the patients, the aneurysm type was saccular, with the distribution of the aneurysm locations consistent with that of the general population. Surgical indications were determined independently by two neurosurgeons. The pterional approach was the most frequent surgical approach, and bypass was used in 7% of surgeries involving clipping or trapping. Aneurysms in the C2 segment of the internal carotid artery were frequently of the blister-like type, and posterior circulation aneurysms often involved arterial branches, leading to the decision that open surgery with bypass was necessary. Surgery was basically performed on the day of onset or the following day (80%), although surgery was performed on day 2–4 with delayed hospital visit due to mild symptom in 22 patients (13%) and delayed surgery on day 5 or later was performed due to vasospasm at the time of referral in 13 patients (7%).

**Table 1:** Baseline characteristics of patients with aneurysmal subarachnoid haemorrhage and surgical procedures

	Number (%)/Median [IQR]
***Patient factor***	
Age, years	57 [47–70]
Female sex	120 (68%)
Comorbidities	
Hypertension	92 (52%)
Dyslipidaemia	33 (19%)
Diabetes mellitus	7 (4%)
History of malignancy	20 (11%)
Active smoking	57 (32%)
Concomitant unruptured aneurysm	38 (22%)
Use of antithrombotic	7 (4%)
mRS 1 or 2 before SAH onset	15 (9%)
***SAH factor***	
WFNS grading	
I	75 (43%)
II	101 (57%)
GCS	
15	75 (43%)
14	75 (43%)
13	26 (15%)
Aneurysm type	
Saccular	163 (93%)
Maximum dome diameter, mm	5.0 [3.9–7.0]
Dissecting	13 (7%)
Maximum length, mm	6.3 [3.2–9.9]
Location of aneurysm	
Acom	46 (26%)
IC-Pcom	44 (25%)
MCA	38 (22%)
IC-Ach	12 (7%)
IC C2	11 (6%)
Distal ACA	6 (3%)
VA	8 (5%)
BA	7 (4%)
Other posterior circulation	4 (2%)
Fisher group 3	157 (89%)
Concomitant ICH	11 (6%)
***Surgical factor***	
Approach	
Pterional	143 (81%)
Interhemispheric	22 (13%)
Suboccipital	11 (6%)
Bypass	14 (7%)
High-flow bypass	7 (50%)
Low-flow bypass	7 (50%)
Day of surgery from onset	
Day 0,1	141 (80%)
Day 2–4	22 (13%)
Day 5 or later	13 (7%)

ACA, anterior cerebral artery; Acom, anterior communicating artery; BA, basilar artery; GCS, Glasgow coma scale; ICH, intracerebral haemorrhage; IC-Pcom, internal carotid artery-posterior communicating artery; IQR, interquartile range; MCA, middle cerebral artery; VA, vertebral artery; WFNS, World Federation of Neurosurgical Societies

Variables are indicated as number (%) and median [interquartile range]

### Primary outcome and postoperative adverse events

At discharge, the mRS score was 0 for 65 (37%), 1 for 30 (17%), 2 for 39 (22%), 3 for 19 (11%), 4 for 19 (11%), 5 for 2 (1%), and 6 for 2 (1%) patients. The overall rate of favourable outcomes was 76%, with 22% of the patients having mRS of 3 or 4 and 2% having mRS of 5 or 6. Among the patients who died, one was an 83-year-old man who experienced postoperative rerupture of a 15-mm internal carotid artery-anterior choroidal artery aneurysm, considered as rapid growth of neck remnant, and his family opted to withdraw from further treatment due to his advanced age. The other patient was a 60-year-old woman undergoing active treatment for breast cancer who experienced severe SAH from a newly ruptured vertebral artery dissecting aneurysm after clipping for an anterior communicating artery aneurysm.

All the postoperative adverse events are summarised in Table 2. Symptomatic cerebral infarction due to surgery occurred in 5% of the patients. Emergent additional surgery for postoperative haemorrhage, cerebral swelling, or aneurysm rerupture was performed for 10 patients (6%). Only 9% of the patients developed DCI, despite 49% having confirmed vasospasm. Epileptic seizures occurred in 21 patients (12%), all of which required antiepileptic drug treatment. Meningitis (requiring extended antibiotic therapy) affected 32 patients (18%). Symptomatic secondary hydrocephalus requiring shunt placement occurred in 43 patients (19%). Pneumonia and electrolyte abnormalities due to cerebral salt-wasting syndrome, diabetes insipidus, and the syndrome of inappropriate antidiuretic hormone secretion were the most common systemic complications. Each occurred in 15 patients (9%) with no associated deaths.

**Table 2:** Details of postoperative and systemic complications after surgery for low-grade SAH

	Total (*N* = 176)	Favourable outcome (mRS 0–2, *N* = 134)	Unfavourable outcome (mRS 3–6, *N* = 42)	*P*-value
*Postoperative complication*				
Symptomatic infarction by surgery	9 (5%)	0	9 (5%)	< 0.001^*^
Oculomotor nerve palsy by surgery	6 (3%)	1 (2%)	5 (4%)	0.674
Postoperative rupture	4 (2%)	1 (1%)	3 (7%)	0.015^*^
Postoperative additional surgery	10 (6%)	3 (2%)	7 (17%)	< 0.001^*^
Total vasospasm	87 (49%)	63 (47%)	24 (57%)	0.252
Delayed cerebral infarction	15 (9%)	9 (6.7%)	6 (14%)	0.125
Epileptic seizure	21 (12%)	8 (6%)	13 (31%)	< 0.001^*^
Meningitis	32 (18%)	18 (13%)	14 (33%)	0.004^*^
Secondary hydrocephalus	43 (19%)	14 (11%)	20 (48%)	< 0.001^*^
*Systemic complication*				
Pneumonia	15 (9%)	7 (5%)	8 (19%)	0.005^*^
CSWS, DI, SIADH	15 (9%)	9 (7%)	6 (14%)	0.125
Liver failure/ cholecystitis	13 (7%)	8 (6%)	5 (12%)	0.200
Heart failure	9 (5%)	5 (4%)	4 (10%)	0.137
Gastrointestinal bleeding	8 (5%)	3 (2%)	5 (12%)	0.009^*^
Urinary tract infection	4 (2%)	2 (1%)	2 (5%)	0.215
Pulmonary embolisation	2 (1%)	1 (1%)	1 (2%)	0.383

CSWS, cerebral salt-wasting syndrome; DI, diabetes insipidus; N, number; SIADH, syndrome of inappropriate antidiuretic hormone secretion

Values are indicated as number (%)

These complications were compared between the favourable outcome group and the unfavorable outcome group at discharge (Table 2). In the unfavorable outcome group, surgery-related complications, including symptomatic infarction, oculomotor nerve palsy, postoperative rerupture, postoperative additional surgery, epileptic seizures, meningitis, and secondary hydrocephalus, were significantly more frequent. Additionally, medical complications, including pneumonia and gastrointestinal bleeding, were also significantly more frequent.

### Analyses of favourable outcomes using background factors and adverse events

In the bivariate analysis, significant negative correlations with favourable outcomes were found for age (continuous, per increasing decade, odds ratio [OR] 0.51, 95% confidence interval [CI] 0.38–0.69, *p* < 0.001) and mRS 1 or 2 before SAH onset (OR 0.32, 95% CI 0.11–0.94, *p* = 0.038). WFNS grade II against grade I did not show statistical significance (*p* = 0.083, Table 3). In the multivariable analysis, age (per increasing decade, OR 0.55, 95% CI 0.40–0.76, *p* < 0.001) was the only significant negative factor for favourable outcomes, making it a key result of this study. ROC curve analysis for age and favourable outcomes showed an area under the curve of 0.737, with the age of 70 years having a sensitivity of 0.866, specificity of 0.500, positive predictive value of 0.847, and negative predictive value of 0.538. Patients aged ≤ 70 years had significantly better outcomes than those aged > 70 years (favourable outcome: 84% vs. 46%, respectively; *p* < 0.001).

**Table 3:** Analysis of baseline factors related to favourable outcomes after surgery for low-grade subarachnoid haemorrhage

	Bivariate		Multivariable	
	OR [95% CI]	*P*-value	OR [95% CI]	*P*-value
*Patient factor*				
Age (continuous, per increasing decade)	0.51 [0.38–0.69]	< 0.001^*^	0.55 [0.40–0.76]	< 0.001^*^
Dichotomised				
Age > 70 years (vs. ≤70 years)	0.16 [0.07–0.34]	< 0.001^*^		
Stratified				
Age > 80 years (vs. <65 years)	0.08 [0.02–0.33]	< 0.001^*^		
Age > 80 years (vs. 65–80 years)	0.23 [0.05–1.00]	0.049^*^		
Age 65–80 years (vs. <65 years)	0.34 [0.16–0.74]	0.007^*^		
Female sex	0.70 [0.32–1.52]	0.371		
Hypertension	0.77 [0.38–1.55]	0.470		
Dyslipidaemia	0.55 [0.24–1.26]	0.161		
Diabetes mellitus	0.22 [0.05–1.01]	0.052	0.35 [0.06–1.93]	0.289
History of malignancy	0.42 [0.16–1.10]	0.079		
Active smoking	2.05 [0.90–4.63]	0.086		
Concomitant unruptured aneurysm	0.85 [0.37–1.93]	0.689		
Use of antithrombotic	0.78 [0.14–4.15]	0.766		
mRS 1 or 2 before SAH onset (vs. mRS 0)	0.32 [0.11–0.94]	0.038^*^	0.52 [0.15–1.84]	0.311
*SAH factor*				
WFNS grade II (vs. grade I)	0.52 [0.25–1.09]	0.083	0.46 [0.20–1.03]	0.060
GCS		0.095		
GCS 13 (vs. GCS 15)	0.36 [0.12–0.90]	0.031^*^		
GCS 14 (vs. GCS 15)	0.62 [0.28–1.37]	0.234		
Fisher group 3 (vs. 1,2)	1.16 [0.39–3.43]	0.791		
Anterior circulation (vs. posterior)	0.16 [0.02–1.21]	0.076	0.16 [0.02–1.45]	0.104
With ICH (vs. without ICH)	0.83 [0.21–3.26]	0.784		
Maximum diameter of aneurysm, mm	1.02 [0.91–1.15]	0.710		
*Operative factor*				
Bypass surgery	0.38 [0.12–1.17]	0.092		
Early surgery at day 0, 1 (vs. delayed surgery after day 2)	0.78 [0.20–1.88]	0.550		

CI, confidence interval; GCS, Glasgow coma scale; ICH, intracerebral haemorrhage; OR, odds ratio; SAH, subarachnoid haemorrhage; WFNS, World Federation of Neurosurgical Societies

*P values < 0.05 are considered significant

The associations between the postoperative adverse events and outcomes are shown in Table 4. In the bivariate analysis, the postoperative events negatively correlated with favourable outcomes, including symptomatic infarction by surgery (*p* = 0.002), postoperative rupture (*p* = 0.047), postoperative additional surgery (*p* = 0.003), epileptic seizure (*p* < 0.001), meningitis (*p* = 0.005), and secondary hydrocephalus (*p* < 0.001). The systemic complications included pneumonia (*p* = 0.009), gastrointestinal bleeding (*p* = 0.019), and urinary tract infection (*p* = 0.047). The development of DCI was not significantly correlated to favourable outcomes. Multivariable analysis identified symptomatic infarction caused by surgery (OR 0.04, 95% CI 0.01–0.27, *p* < 0.001), epileptic seizure (OR 0.11, 95% CI 0.03–0.36, *p* < 0.001), secondary hydrocephalus (OR 0.10, 95% CI 0.04–0.27, *p* < 0.001), and urinary tract infection (OR 0.06, 95% CI 0.01–0.74, *p* = 0.028) as significant negative factors.

**Table 4:** Risk analysis of complication factors related to favourable outcomes after surgery for low-grade subarachnoid haemorrhage

	Bivariate		Multivariable	
	OR [95% CI]	*P*-value	OR [95% CI]	*P*-value
*Postoperative surgical complications*				
Symptomatic infarction by surgery	0.08 [0.02–0.38]	0.002^*^	0.04 [0.01–0.27]	< 0.001^*^
Oculomotor nerve palsy by surgery	1.59 [0.18–14.00]	0.677		
Postoperative rupture	0.10 [0.01–0.97]	0.047^*^		
Postoperative additional surgery	0.11 [0.03–0.47]	0.003^*^	0.19 [0.03–1.10]	0.064
Total vasospasm	0.67 [0.33–1.34]	0.253		
Delayed cerebral infarction	0.43 [0.14–1.29]	0.134		
Epileptic seizure	0.14 [0.05–0.37]	< 0.001^*^	0.11 [0.03–0.36]	< 0.001^*^
Meningitis	0.31 [0.14–0.70]	0.005^*^		
Secondary hydrocephalus	0.13 [0.06–0.29]	< 0.001^*^	0.10 [0.04–0.27]	< 0.001^*^
*Systemic complications*				
Pneumonia	0.23 [0.08–0.69]	0.009^*^		
CSWS, DI, SIADH	0.54 [0.19–1.55]	0.251		
Liver failure/ cholecystitis	0.47 [0.14–1.52]	0.208		
Heart failure	0.37 [0.09–1.44]	0.151		
Gastrointestinal bleeding	0.17 [0.04–0.74]	0.019^*^		
Urinary tract infection	0.10 [0.01–0.97]	0.047^*^	0.06 [0.01–0.74]	0.028^*^
Pulmonary embolisation	0.31 [0.02–5.04]	0.409		

CI, confidence interval; ICH, intracerebral haemorrhage; OR, odds ratio; PA, pterional approach; WFNS, World Federation of Neurosurgical Societies

*P values < 0.05 are considered significant

### Prognostic risk classification by recursive partitioning analysis

**Fig. 1:**
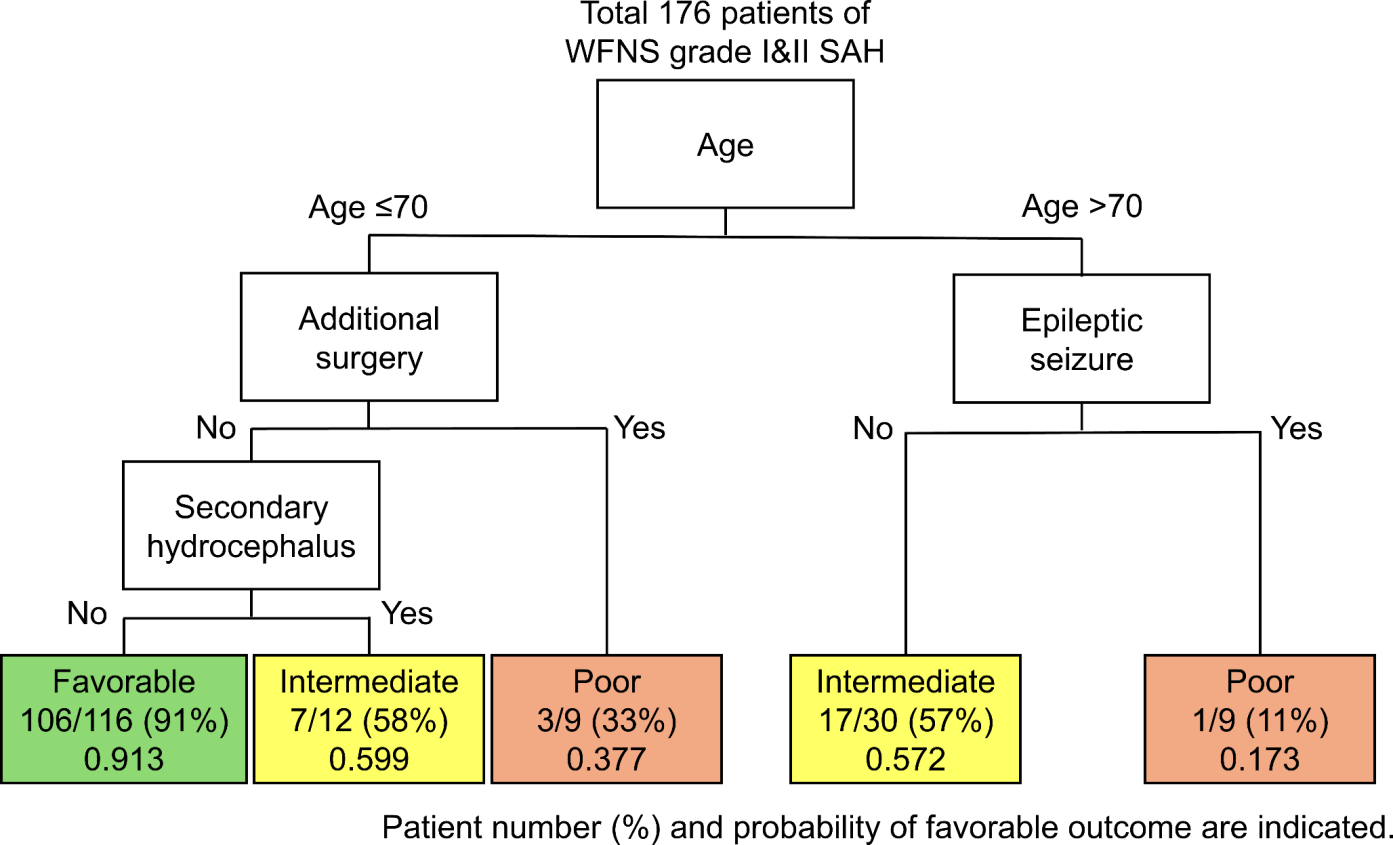
Recursive partitioning analysis of favourable outcomes (mRS 0–2 at discharge) in a predictive model with three outcomes based on age dichotomisation using the Youden index for 70 years

Starting with the age of 70 years as a significant partitioning factor, further optimal partitioning created the risk classification using RPA as shown in Fig. 1. The favourable prognosis group included patients aged ≤ 70 years who did not require additional surgery or did not have secondary hydrocephalus and had a favourable outcome probability of 0.913. The intermediate prognosis group included patients aged ≤ 70 years who did not require additional surgery but had secondary hydrocephalus and patients aged > 70 years without epileptic seizures (favourable outcome probabilities of 0.599 and 0.572, respectively). The poor prognosis group included patients aged ≤ 70 years requiring additional surgery and those aged > 70 years with epileptic seizures (favourable outcome probabilities of 0.377 and 0.173, respectively). Nominal logistic regression for favourable outcomes across these groups showed significant differences, with ORs (95% CI) for favourable vs. intermediate, intermediate vs. poor, and favourable vs. poor of 7.95 (3.26–19.38, *p* < 0.001), 4.67 (1.31–16.59, *p* = 0.017), and 37.10 (10.25–134.31, *p* < 0.001), respectively (Table 5).

**Table 5:** Prognostic model for predicts favourable outcomes after surgery for low-grade subarachnoid haemorrhage

Prognosis group	OR [95% CI]	*P*-value
Favourable (vs. intermediate)	7.95 [3.26–19.38]	< 0.001^*^
Intermediate (vs. poor)	4.67 [1.31–16.59]	0.017^*^
Favourable (vs. poor)	37.10 [10.25–134.31]	< 0.001^*^

CI, confidence interval; OR, odds ratio

*P values < 0.05 are considered significant

## Discussion

This study demonstrated that age was a stronger prognostic factor than WFNS grading for patients with low-grade SAH (WFNS grade I/II) undergoing microsurgical clipping. Using 70 years as a cutoff, the study developed a clinically applicable risk evaluation method, dividing the patients into three groups (favourable, intermediate, and poor) based on age and postoperative adverse events.

Initially, this study investigated the association between background factors related to patients, disease, and surgery with favourable outcomes (mRS 0–2 at discharge). The age at onset was the sole prognostic predictor, highlighting its importance in the prognosis of low-grade SAH. The WFNS grading system has been widely used since its introduction in 1988 to classify prognoses based on the level of consciousness at SAH onset [34], but some studies have reported no significant prognostic difference between WFNS grades [27, 32, 38]. Low-grade SAH (WFNS grades I and II) is generally considered to have a favourable prognosis, but distinguishing between grades can be challenging in older patients with pre-existing cognitive decline. Zijlmans et al. identified age as a background factor associated with unfavourable outcomes (mRS 3–6) at 6 months after treatment in 132 patients with WFNS grade I aneurysmal SAH [45]. Conversely, Hori et al. found no background factors, including age, associated with unfavourable outcomes (Glasgow Outcome Scale 1–3) at discharge in 171 patients with WFNS grade I and II SAH, making the findings controversial [13]. These studies included both surgical and endovascular treatments and had heterogeneous cohorts. In our study, age was a stronger prognostic factor for the surgical cohort, suggesting that the higher invasiveness of open surgery may be more impactful on the outcomes of older patients.

Using the age of 70 years as a partitioning factor, the RPA in this study showed optimal partitioning based on surgical-related complications in patients younger than 70 years and post-surgical epileptic seizures in those older than 70 years for the prediction of favourable outcomes. In the elderly group, postoperative seizures were particularly detrimental, significantly increasing the likelihood of unfavourable outcomes at discharge. This suggests that controlling seizures in older patients is more difficult, possibly due to underlying age-related neurological changes that make seizure management less effective. Moreover, the elderly population tends to experience faster declines in activities of daily living (ADL) following complications like seizures, further compounding the negative impact on recovery. The association between poor outcomes and epilepsy at discharge has been reported, suggesting the need for careful surgical techniques to minimise complications [3, 8, 11, 12, 14, 19, 25, 35]. Therefore, more intensive postoperative seizure monitoring and management should be prioritized in older patients to minimize this risk. Although the use of prophylactic antiepileptic drugs in patients without preoperative seizures is debated [44], our findings suggest that it might be worth considering their use in elderly patients, given the significant impact of postoperative seizures on outcomes.

In contrast, younger patients were less affected by seizures but were more vulnerable to unfavourable outcomes if additional surgeries were required. The need for additional procedures, typically due to postoperative haemorrhage, brain swelling, or aneurysm rerupture, directly contributed to poor outcome group. Needless to say, these findings highlight the critical importance of meticulous surgical technique to prevent such complications. Additionally, in cases where significant brain swelling is anticipated, early decompressive craniotomy should be considered as a proactive measure to reduce the risk of further complications and improve recovery prospects. While surgical precision is crucial for all patients, our findings suggest that it may be particularly essential in preventing reoperation-related complications in younger patients.

Secondary hydrocephalus is a known predictor of poor outcomes for aneurysmal SAH, and this study confirmed its significance in patients under 70 years with low-grade SAH [1, 29, 42]. Previous studies have identified age as a risk factor for shunt-dependent hydrocephalus and its correlation with poor outcomes and quality of life [1, 29, 41]. The association between hydrocephalus and prognosis in younger patients in this study is noteworthy, although the outcome measurement was mRS at discharge, which may not fully capture long-term recovery.

This study has a few limitations. Firstly, it was a single-centre retrospective study, and selection bias may have occurred. Secondly, the outcome measurement was mRS at discharge, and long-term follow-up could not be conducted. Thirdly, the classification model has not been validated in a separate cohort. Multicentre prospective studies with long-term follow-up data are needed for further validation.

## Conclusion

Age was a more significant prognostic factor than WFNS grading in patients with WFNS grade I/II aneurysmal subarachnoid haemorrhage undergoing microsurgical clipping. The developed prognostic model with an age cut-off of 70 years and postoperative complications provides a practical tool for predicting outcomes and guiding treatment strategies. In that model, precise microsurgeries with fewer complications determined favourable outcomes beyond the WFNS grade for younger patients, while postoperative intensive seizure management could be meaningful to avoid poor outcomes for older patients.

## Data Availability

No datasets were generated or analysed during the current study.
